# The integrated stress response in apicomplexan and trypanosomatid parasites: balancing survival and pathogenesis

**DOI:** 10.3389/fimmu.2026.1804980

**Published:** 2026-03-31

**Authors:** Ikram Hammi, Dounia Darif, Damien Arnoult

**Affiliations:** 1 Institut National de la Santé et de la Recherche Médicale, Unité Mixte de Recherche, Santé, Ministère de la Défense INSERM UMR-S-MD 1197, Ministère des Armées, Université Paris-Saclay, Villejuif, France; 2 Health and Environment Laboratory, Aïn Chock Faculty of Sciences, Hassan II University of Casablanca, Casablanca, Morocco; 3 INSERM-U1124, Université Paris Cité, Paris, France

**Keywords:** Integrated Stress Response (ISR), eIF2a, host-parasite interaction, unfolded protein response (UPR), apicomplexa, trypanosomatida

## Abstract

Parasites rely on their hosts for survival and replication and therefore face major challenges as they transition between hosts and encounter hostile microenvironments. Over evolution, these organisms have developed complex mechanisms to rapidly adapt to environmental fluctuations and to exploit host cellular machinery for persistence and dissemination. Among the pathways parasites deploy to cope with stress is the Integrated Stress Response (ISR), a conserved mechanism present in both parasites and their hosts, yet built on partially distinct molecular components. This shared but divergent pathway constitutes a critical battleground that reflects host–parasite coevolution, where host-protective responses intersect with parasite adaptive strategies. This review summarizes the current understanding of how protozoan parasites engage their own ISR-like programs to adapt to harsh environments and how they modulate host ISR signaling to establish and maintain successful infections.

## 1 Introduction

The Integrated Stress Response (ISR) is a cellular surveillance system that detects perturbations in homeostasis, including amino acid deprivation, viral infection, oxidative stress, endoplasmic reticulum (ER) stress, and genotoxic damage. In mammalian cells, these stress signals converge on eukaryotic translation initiation factor 2 alpha (eIF2α), a central regulator of translation initiation. Phosphorylation of eIF2α attenuates global protein synthesis while promoting selective translation of specific transcripts encoding stress-responsive transcription factors such as activating transcription factor 4 (ATF4). The unfolded protein response (UPR) represents a specialized arm of this surveillance system, activated specially when misfolded or unfolded protein accumulates in the ER lumen, which overwhelms its folding capacity (1). Cells respond by inducing gene-expression programs that restore homeostasis through enhanced proteostasis, antioxidant defenses, and autophagy. If these adaptive mechanisms prove insufficient, cells can undergo apoptotic death (2).

Protozoan parasites also possess ISR-like stress response pathways that enable rapid adaptation to environmental challenges encountered throughout their life cycles. These pathways, while often underexplored compared to those of mammalian hosts, are functionally specialized and can differ substantially in their molecular components. Parasites such as *Plasmodium*, *Toxoplasma*, *Leishmania*, and *Trypanosoma* species use intrinsic stress machinery to sense and respond to nutrient limitation, temperature fluctuations, oxidative stress, and other hostile conditions (3, 4). Moreover, through secreted effector molecules, parasites can modulate host ISR signaling by targeting key pathway components, thereby dampening protective responses or inducing controlled stress conditions that favor immune evasion and intracellular persistence (5).

Although host stress pathways have been extensively studied, and parasite stress-adaptation mechanisms increasingly recognized, how parasites and hosts influence each other’s stress responses remains incompletely understood. Defining this cross-talk is critical to understand how intracellular parasites exploit ancient stress-sensing pathways to survive and establish chronic infection. This gap highlights the interplay between parasite-intrinsic stress responses and parasite-mediated manipulation of host stress pathways, creating multilayered regulatory networks in which parasites must balance their own survival with maintenance of host-cell viability.

This review synthesizes current understanding of how intracellular parasites employ the ISR, UPR, and mitochondria-linked stress responses to adapt to hostile environments and establish successful infections while evading host surveillance. We examine (i) the molecular mechanisms by which parasites sense environmental changes and coordinate developmental transitions through parasite-intrinsic eIF2α kinases (4, 6); (ii) strategies by which parasites manipulate host ER and mitochondrial stress pathways to suppress antimicrobial responses and promote infected-cell survival (7, 8); and (iii) evolutionary innovations that have shaped parasite stress-response machinery into specialized tools for host–parasite interactions. By highlighting these molecular dialogues between ancient stress-sensing pathways and parasitic adaptations, this review identifies therapeutic opportunities for selective interventions that exploit fundamental differences in stress-response architecture between parasites and their hosts.

## 2 The integrated stress response

The ISR is an evolutionarily conserved eukaryotic program that coordinates adaptation to diverse environmental challenges. It can be triggered by external stresses such as hypoxia, nutrient deprivation (amino acids or glucose), and viral infection, as well as intrinsic stresses such as ER proteotoxicity caused by accumulation of misfolded proteins.

**Figure 1:**
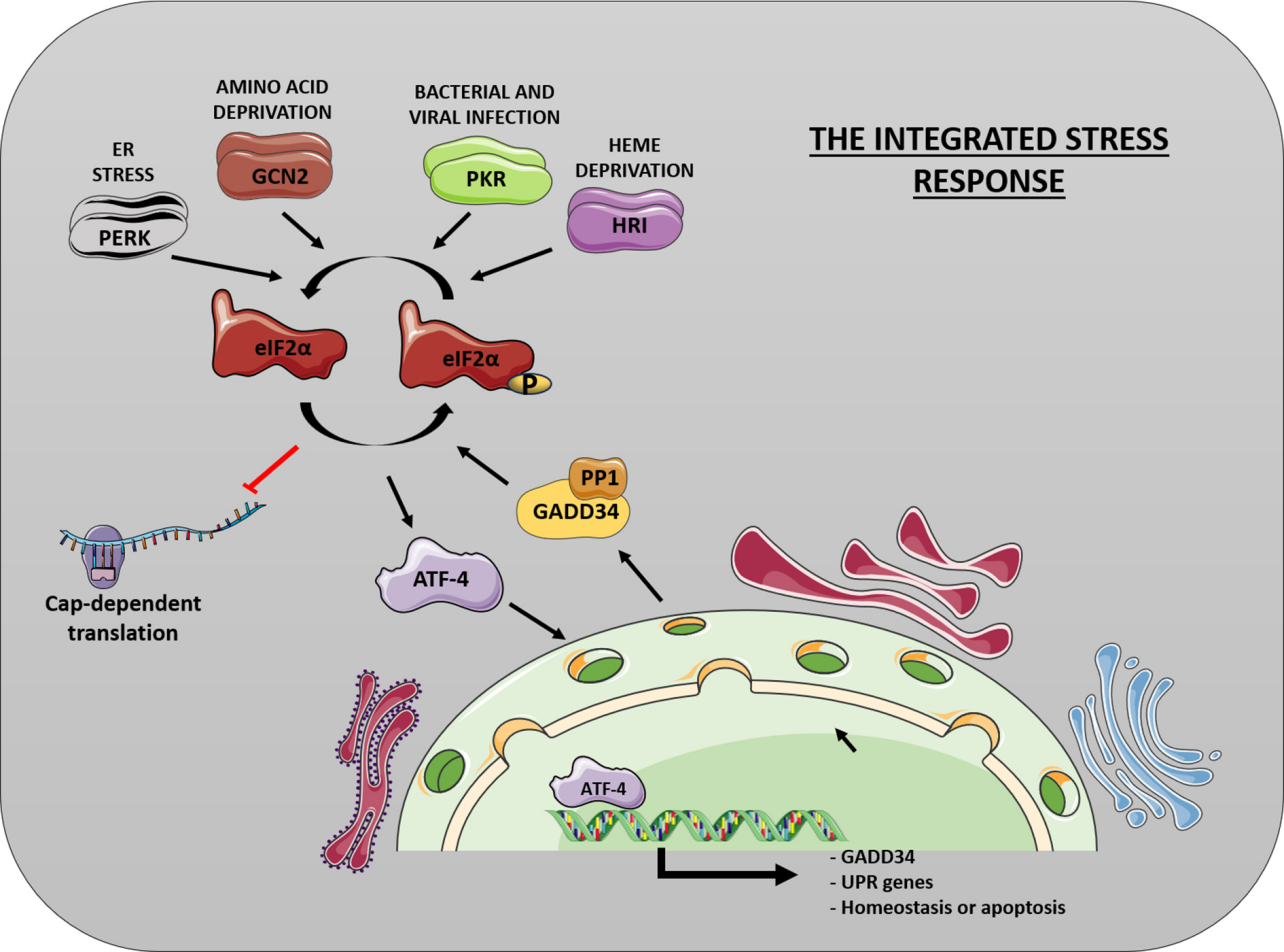
Molecular architecture of the Integrated Stress Response (ISR). Diverse stress stimuli activate specific eIF2α kinases (PERK, PKR, GCN2, HRI), which homodimerize and undergo trans-autophosphorylation to achieve catalytic competence. Once activated, these kinases phosphorylate eIF2α, leading to broad translational repression of cap-dependent mRNAs. Conversely, transcripts harboring upstream open reading frames (uORFs) display preferential translation via cap-independent initiation. These selectively translated mRNAs encode essential stress effectors, most notably ATF4, a transcription factor that assembles into dimeric complexes to orchestrate adaptive gene expression programs, ultimately promoting cellular stress recovery and homeostatic restoration. This figure was drawn using PowerPoint, incorporating images from the Smart Servier Medical Art library (smart.servier.com).

At the molecular level, the ISR converges on phosphorylation of eIF2α at serine 51. This phosphorylation is catalyzed by one of four stress-responsive kinases: PERK (activated by ER stress), GCN2 (amino acid deprivation), PKR (viral double-stranded RNA), and HRI (heme deficiency, primarily in erythroid cells). These kinases are typically activated through dimerization and autophosphorylation. eIF2α phosphorylation triggers a global reduction in protein synthesis, while paradoxically promoting selective translation of specific transcripts, most notably ATF4. ATF4 coordinates adaptive gene-expression programs that facilitate recovery from stress (**Figure 1**).

Termination of the ISR occurs through dephosphorylation of eIF2α by protein phosphatase 1 (PP1) in complex with regulatory subunits: growth arrest and DNA damage-inducible protein 34 (GADD34/PPP1R15A), induced during stress as part of a negative feedback loop, and constitutive repressor of eIF2α phosphorylation (CReP/PPP1R15B), which maintains basal eIF2α dephosphorylation under unstressed conditions, thereby restoring normal protein synthesis (**Figure 1**).

When stress is excessive or persistent and exceeds the adaptive capacity of the ISR, signaling can shift from pro-survival to pro-apoptotic. In this context, the duration and magnitude of eIF2α phosphorylation, ATF4 output, and interactions with additional pathways determine whether cells adapt or undergo programmed death (2, 9).

## 3 Regulation of eIF2α kinases

The four mammalian eIF2α kinases share a common substrate yet respond to distinct cellular stressors through specialized sensing domains and regulatory mechanisms. Understanding how each kinase is activated and regulated clarifies context-specific translational control (10).

### 3.1 GCN2

General control nonderepressible 2 (GCN2) is highly conserved from yeast to mammals and provides insight into ancestral stress-detection systems (2). In yeast, GCN2 senses amino acid scarcity through a histidyl-tRNA synthetase–related domain that binds uncharged tRNAs that accumulate during nutrient limitation (11). GCN2 can also be activated during prolonged glucose deprivation in cancer cells, potentially reflecting secondary consequences of altered amino acid metabolism as cells shift substrates. UV irradiation has been reported to activate GCN2 in mouse embryonic fibroblasts and human keratinocytes through incompletely defined pathways. Proposed mechanisms include UV-mediated tRNA-GCN2 crosslinking or accelerated arginine depletion downstream of nitric oxide synthase activity (12).

### 3.2 PKR

Protein kinase R (PKR) primarily functions as a viral RNA sensor, recognizing double-stranded RNA generated during infection and contributing to antiviral immunity. PKR dimerizes through its C-terminal kinase domain, undergoes autophosphorylation (including at threonine 446), and suppresses both viral and cellular protein synthesis via eIF2α phosphorylation. PKR can also be activated independently of dsRNA by diverse cellular stressors, including oxidative damage, ER proteotoxic stress, growth factor withdrawal, inflammatory cytokines, bacterial invasion, ribosomal stress, stress granule assembly, and exposure to polyanions such as heparin. Caspase-mediated cleavage can modulate PKR during early apoptosis, linking translational repression to programmed cell death (13, 14).

### 3.3 PERK and the UPR

ER stress arises when ER functions such as protein folding and maturation, lipid/sterol biosynthesis, and calcium homeostasis, are compromised. In mammals, ER stress triggers the unfolded protein response (UPR), coordinated by three ER transmembrane sensors: PERK, IRE1, and ATF6. These sensors detect misfolded proteins and activate overlapping yet distinct programs that expand ER capacity and promote ER-associated degradation (ERAD) (15, 16).

IRE1 and PERK are activated by oligomerization and phosphorylation, whereas ATF6 requires Golgi translocation and proteolytic processing. Activated IRE1 catalyzes unconventional splicing of XBP1 mRNA, generating a frameshifted transcript that is translated into the highly active transcription factor XBP1s protein. XBP1s induces chaperones, ERAD factors, and autophagy-associated components; unspliced XBP1 (XBP1u) can antagonize XBP1s and has been reported to protect endothelial cells from oxidative injury through Akt phosphorylation and NRF2-dependent HO-1 induction when complexed with HDAC3. The PERK arm phosphorylates eIF2α at serine 51, suppressing global translation while enhancing selective synthesis of ATF4. ATF4 promotes transcriptional outputs, including the key pro-apoptotic mediator CHOP/DDIT3, and antioxidant defense genes. Together, these branches calibrate adaptation versus apoptosis depending on stress severity and duration (2, 17).

### 3.4 HRI and mitochondria-to-ISR signaling

Heme-regulated inhibitor kinase (HRI) is enriched in erythroid cells, where it coordinates globin synthesis with heme availability to prevent toxic globin aggregation under heme/iron limitation. HRI activation involves dimerization and autophosphorylation and is modulated by heme binding through motifs located in the N-terminus and in the kinase insertion loop. Binding at the insertion site is dynamic and suppresses kinase activity. Heme can maintain HRI in an inhibited state by promoting intermolecular disulfide bridges that lock dimers; under heme depletion, non-covalent dimerization yields active complexes (9).

Beyond heme scarcity, HRI responds to arsenite-mediated oxidative damage, heat stress, osmotic stress, proteasome dysfunction, and nitric oxide exposure via heme-independent mechanisms involving chaperones such as HSP90 and HSP70, although detailed activation pathways remain incompletely defined (9, 16).

**Figure 2:**
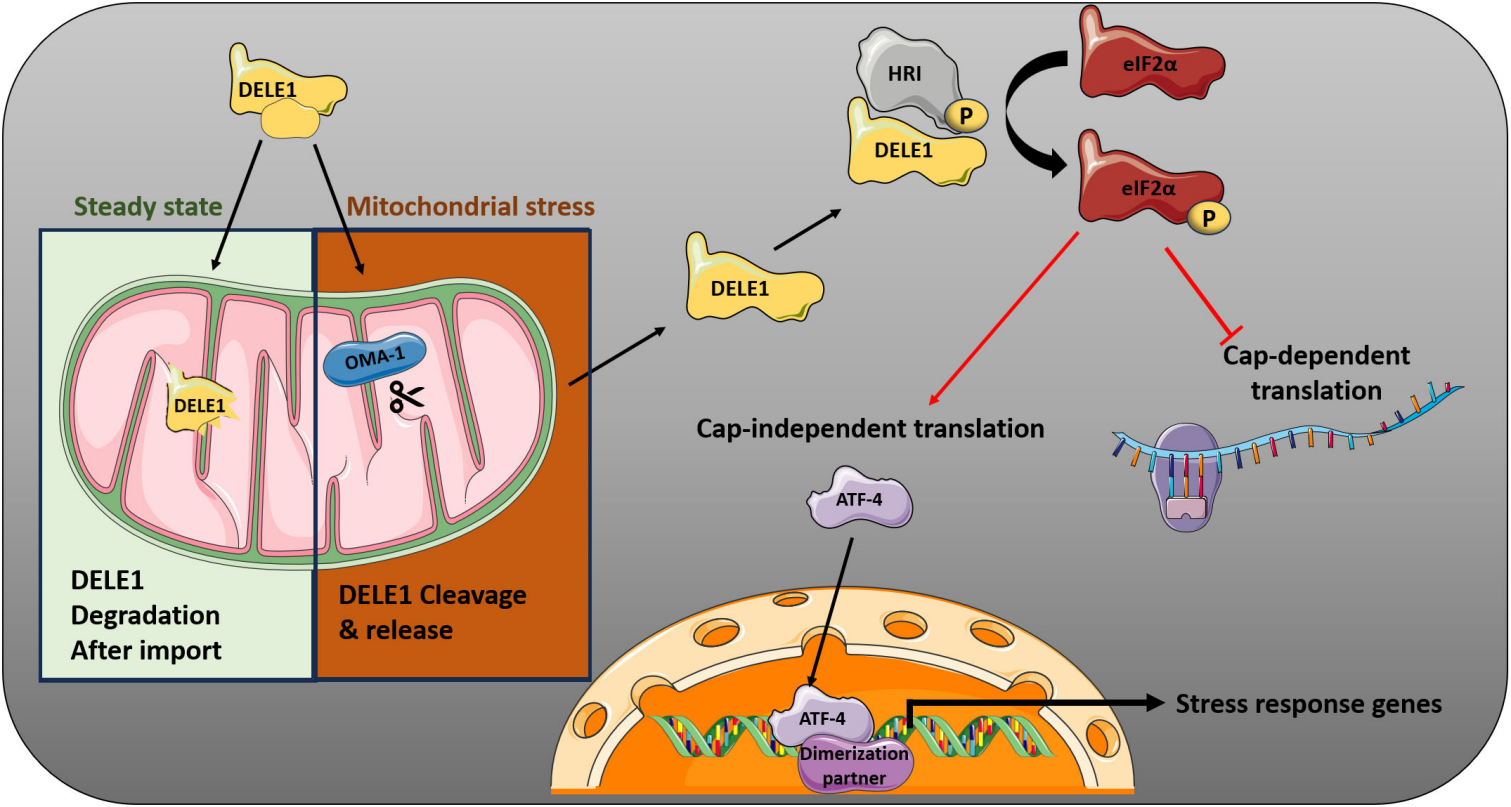
Mitochondria-to-ISR signaling via the OMA1–DELE1–HRI axis. Under steady-state condition and soon after import, DELE1 is degraded by a mitochondrial matrix-resident protease. But during Mitochondrial perturbation, the protease OMA1, which resides in the inner mitochondrial membrane, is activated. OMA1 cleaves DELE1, generating a truncated form DELE-1s that is released into the cytosol. There, DELE1s binds to HRI and activates it. Activated HRI then phosphorylates eIF2α, initiating ISR. This figure was drawn using PowerPoint, incorporating images from the Smart Servier Medical Art library (smart.servier.com).

Mitochondrial dysfunction can engage the ISR via the DELE1/HRI axis (**Figure 2**). DELE1, a key component of HRI-mediated ISR activation, can be processed by multiple proteases depending on parameters such as cell type, stimulus duration, and the nature of the mitochondrial stress. The protease OMA1 was initially identified as the primary enzyme responsible for DELE1 cleavage under mitochondrial stress conditions (9). More recent work has identified HtrA2 as an additional protease capable of processing DELE1 during mitochondrial protein import stress (MPIS), thereby increasing the regulatory complexity of this pathway. Although both OMA1 and HtrA2 can process DELE1, their relative contributions appear to be context-dependent and may vary across cell types and stress conditions (18).

DELE1 functions as a multimodal sensor of mitochondrial protein import and processing defects. When protein import through the TIM23 complex is impaired, full-length DELE1 (L-DELE1) accumulates in the cytosol and retains the ability to bind and activate HRI independently of proteolytic cleavage. This observation suggests that DELE1 can signal mitochondrial stress through multiple molecular forms, including cleaved short DELE1 (S-DELE1), an intermediate very short form (VS-DELE1), and cytosolic full-length DELE1, potentially arising from distinct import perturbations. Regardless of the molecular form, oligomerization of DELE1 into an octameric complex is essential for efficient HRI activation, acting as a scaffold that enhances kinase activity. These cytosolic DELE1 oligomers promote HRI activation, leading to eIF2α phosphorylation and subsequent ATF4-dependent transcriptional responses. The downstream outcomes depend on the nature, duration, and intensity of mitochondrial stress, as well as the cellular context (17, 18).

## 4 SIFI and termination of the ISR

ISR resolution involves two complementary routes: (i) PP1-dependent eIF2α dephosphorylation mediated by PPP1R15A (GADD34) and PPP1R15B (CReP), and (ii) ubiquitin-dependent degradation of stress-response components by the SIFI complex (UBR4/KCMF1). SIFI acts as a stress-sensitive silencing module that detects mitochondrial precursor proteins accumulating under stress and proteins released from compromised mitochondria. These stress-associated proteins compete with HRI for SIFI binding, thereby delaying ISR silencing until stress is adequately resolved. Genetic evidence supports the physiological importance of SIFI: UBR4 deletion causes embryonic lethality in mice, and UBR4 mutations in humans have been associated with ataxia and early-onset dementia. Together, these termination mechanisms ensure that ISR signaling is reversible and appropriately resolved to restore homeostasis (19, 20).

## 5 ISR as an ancestral survival mechanism in parasites

The ISR is an ancient stress-sensing mechanism that intracellular parasites have conserved and repurposed to survive dramatic environmental changes across complex life cycles. Whereas mammals rely on four canonical eIF2α kinases, protozoan parasites possess distinct and often parasite-specific kinase repertoires that enable translational control during differentiation, stress adaptation, and host interaction. GCN2-like kinases are widely conserved, PERK orthologues occur in metazoans, and HRI exists even in some fungi such as *Schizosaccharomyces pombe* (4, 21). Many parasites encode eIF2α kinases with limited orthology to mammalian counterparts, suggesting that these organisms have undergone substantial lineage-specific innovation in stress-sensing mechanisms. These innovations operate at both the parasite and host levels, as detailed in **Tables 1**, **2**.

**Table 1:** eIF2α kinase orthologs in Apicomplexa and Trypanosomatida: subcellular localisation, mammalian ortholog, and accession numbers.

	Species	Kinases	Subcellular localisation	Mammalian ortholog	Accession numbers	Key references
APICOMPLEXA	*P. falciparum*	IK1 (PfeIK1)	Cytoplasm (blood-stage parasites)	GCN2	PF14_0114 (PlasmoDB)	(55–57)
		IK2 (PfeIK2)	Cytoplasm (salivary gland sporozoites)	GCN2-like	PFA0380w (PlasmoDB)	
		PK4 (PfPK4)	Endoplasmic reticulum membrane	PERK/HRI-like	PFF1370 (PlasmoDB)/XP_001348597.1 (Uniprot)	
	*T. gondii*	TgIF2K-A	Endoplasmic reticulum membrane	PERK	TGME49_025340 (ToxoDB)	(6, 58)
		TgIF2K-B	Cytoplasm	HRI-like	TGME49_270240 (ToxoDB)	
		TgIF2K-C	Cytoplasm (intracellular parasites)	GCN2-like	TGME49_204100 (ToxoDB)	
		TgIF2K-D	Cytoplasm (intra and extracellular parasites)	GCN2	TGME49_119610 (ToxoDB)/AED01979.1 (NCBI)	
TRYPANOSOMATIDA	*T.cruzi*	TcK1	Cytoplasm (epimastigotes)	GCN2	NMZO01000706.1 (TriTryp)	(21, 30)
		TcK2	Endosomal membrane	PERK	TcCLB.506559.129 (TriTrypDB)	
		TcK3	Unknown	HRI-like	ADWP02013222 (TriTrypDB)	
	*L. donovani*	LdeK1	Cytoplasm	GCN2	AKG62099.1 (NCBI)	(32)

**Table 2:** ISR activation and inhibition in apicomplexa and trypanosomatida: triggers, and molecular pathways.

	Species	ISR effect	Trigger/stimulus	Molecular pathway	Key references
APICOMPLEXA	*P. falciparum/P. berghei*	Triggers ISR	Amino acid starvation (blood-stage asexual parasites)	IK1 (GCN2-like) → eIF2α-P (Ser59) → global translational attenuation → stress adaptation of blood-stage parasites	(23, 55, 57, 59, 60)
		Triggers ISR	Sporozoite latency (mosquito salivary glands)	IK2 (UIS1) → eIF2α-P → global translation repression → mRNA granule formation → maintenance of sporozoite dormancy	
		Triggers ISR	PK4 (PERK/HRI-like) →eIF2α-P (Ser59) → global translational arrest → schizont maturation and gametocyte development	PK4 (PERK/HRI-like) → eIF2α-P (Ser59) →global translational arrest →schizont maturation and gametocyte development	
	*P. berghei*	Inhibits ISR	Sporozoite-to-liver stage transition (host injection)	UIS2(PP2C/PPM phosphatase) → eIF2α dephosphorylation → translation de-repression → liver stage development	
		Triggers ISR (host cell)	Hepatocyte infection by sporozoites	Parasite-induced ER stress in host hepatocytes → XBP-1 mRNA splicing (IRE1 branch of UPR) → phospholipid synthesis → promotes liver stage replication	
	*T. gondii*	Triggers ISR	ER stress (unfolded protein accumulation)	TgIF2K-A (PERK-like, ER-resident) → TgIF2α-P (Ser71) → global translation attenuation →tachyzoite to bradyzoite differentiation, tissue cyst formation	(6, 58, 61, 62)
		Triggers ISR	Oxidative stress (host macrophage ROS)	TgIF2K-B(HRI-like, cytoplasmic) → TgIF2α-P → translational control → catalase/antioxidant response → limits replication rate (promotes persistent infection)	
		Triggers ISR	Glutamine/amino acid deprivation (intracellular parasites)	TgIF2K-C (GCN2-like) → TgIF2α-P → translational attenuation → arginine transporter CAT1 upregulation in host → nutrient acquisition by parasite	
		Triggers ISR	Extracellular stress/nutrient deprivation after host cell egress	TgIF2K-D(GCN2-like)→TgIF2α-P → translational repression → enhanced extracellular parasite viability while searching for new host cell	
TRYPANOSOMATIDA	*T. cruzi*	Triggers ISR	Nutritional stress/amino acid starvation (epimastigote stage in insect midgut)	TcK1(GCN2-like) → eIF2α-P → stress granule (DHH1/PABP1) formation → translational control →modulates metacyclogenesis	(21, 63)
		Triggers ISR	Heme depletion in insect midgut (post blood meal digestion)	TcK2(PERK-like, endosomal) → eIF2α-P → global translation attenuation→ metacyclogenesis into infective trypomastigotes	
		Inhibits ISR	Heme availability (proliferating epimastigotes)	Heme binds to TcK2 kinase domain → TcK2 inhibition → reduced eIF2α-P → maintained translation → supports epimastigote proliferation	
		Triggers ISR	Unknown stress (TcK3 ortholog in T. brucei responds to undefined signals)	TcK3 (HRI-like, putative) → eIF2α-P → translational arrest → programmed stress-induced cell death (via TBP phosphorylation in T. brucei ortholog)	
	*L. donovani*	Triggers ISR	Nutrient starvation, acidic pH, heat stress (promastigote in sandfly gut/differentiation signals)	LdeK1 (GCN2-like, cytoplasmic) → eIF2α-P → G1 cell-cycle arrest →translational reprogramming →metacyclogenesis (also implicated in immune modulation (pro-inflammatory response))	(32, 64, 65)
	*L. infantum/L. donovani*	Triggers ISR	Temperature shift + acidic pH stress (promastigote-to-amastigote differentiation inside macrophage phagolysosome)	PERK-like kinase (K2 homolog, ER-resident) → eIF2α-P → global translation decrease → amastigote differentiation; delayed in absence of kinase	
	*L. amazonensis*	Triggers ISR (host cell)	*Leishmania* infection of host macrophages	Parasite activates host PKR → eIF2α-P → IFN-1 modulation (TLR2/PKR-dependent) → immune evasion and promotion of intracellular parasite survival	

### 5.1 *Plasmodium* species

*Plasmodium* spp. are vector-transmitted apicomplexan parasites responsible for malaria, requiring rapid adaptation between mosquito vectors and mammalian hosts, including hepatocytes that support development and replication (22, 23). *Plasmodium* harbors three eIF2α kinases (IK1, IK2, and PK4). IK2 maintains sporozoite latency in mosquito salivary glands via sustained eIF2α phosphorylation but upon transmission, dephosphorylation enables resumption of translation. IK1 (GCN2-like) responds to amino acid starvation and influences the erythrocytic cycle, while PK4 (PERK-like) contributes to schizont and gametocyte development (24).

During gametocyte-to-ookinete differentiation, oxidative stress correlates with increased eIF2α phosphorylation (6–24 h post-activation), reduced translation, and accumulation of immature ookinetes during heightened metabolic demand. This response is associated with induction of ER chaperones (GRP78/BiP, PDI) and antioxidant enzymes such as superoxide dismutase, supporting a role for the ISR in coordinating maturation under stress (25).

### 5.2 Toxoplasma gondii

*Toxoplasma gondii* infects most warm-blooded vertebrates and persists as dormant tissue cysts. While often asymptomatic, it can cause severe disease in immunocompromised patients and during congenital infection (6, 26). Like other eukaryotes, *T. gondii encodes a functional ortholog of eIF2α, TgIF2α, which serves as the central regulatory target of the parasite’s integrated stress response. TgIF2α is phosphorylated during bradyzoite differentiation and under extracellular stress conditions such as amino acid limitation* (27). Environmental changes such as temperature shifts, alkaline pH, chemical stressors, and mitochondrial inhibition, can trigger tachyzoite-to-bradyzoite conversion through TgIF2α phosphorylation.

Multiple parasite kinases contribute to these outputs. TgIF2K-A (ER-localized, PERK-like) reduces translation under ER stress, TgIF2K-B (cytosolic) responds to oxidative stress and supports pathogenicity, and TgIF2K-D (GCN2-like) is activated by arginine deprivation and upregulates arginine transporters, although it is dispensable for entry into nutritional hibernation-like states (3, 6). TgIF2K-B deficiency impairs stress sensing and growth adaptation, emphasizing its role in growth plasticity.

### 5.3 Trypanosoma

*Trypanosoma* spp. cause multiple neglected tropical diseases and undergo extensive remodeling across insect and mammalian stages (28, 29). They encode three putative eIF2α kinases (K1–K3) with distinct localizations and roles. In *T. cruzi*, TcK1 (GCN2-like) influences stress responses and development, apparently partly independent of eIF2α phosphorylation, possibly via the eIF4F complex. Under nutritional stress, TcK1 supports stress granule assembly and survival (21, 30). TcK2 (PERK-like topology) localizes to endosomal membranes and responds to heme deprivation. Under heme sufficiency, imported heme inhibits TcK2, permitting translation. But under heme deprivation, activated TcK2 phosphorylates eIF2a, which suppresses translation and promotes metacyclogenesis. TcK2 knockout alters heme/ROS homeostasis and reduces infectivity (3, 30).

In *T. brucei*, TbeIF2αK2 localizes to the flagellar pocket/early endocytic vesicles. ER stress can induce spliced leader silencing (SLS), characterized by global mRNA reduction, translation inhibition, and a programmed death pathway proposed to eliminate unfit parasites (3).

### 5.4 Leishmania

*Leishmania* spp. transition from promastigotes in sandflies to intracellular amastigotes in macrophage phagolysosomes (31). *L.donovani* encodes LdeK1, a GCN2-like kinase activated by nutrient starvation. LdeK1 phosphorylates eIF2α at a non-canonical Thr166 residue and induces G1 arrest, promoting promastigote-to-amastigote differentiation *(*32). Dominant-negative LdeK1 mutants fail to phosphorylate eIF2α during starvation, cannot achieve G1 arrest, and display impaired differentiation (3, 32). Similar nutrient-coupled GCN2 activation during differentiation has been observed in other eukaryotes, supporting broad conservation of this strategy (33).

## 6 SIFI-like silencing and UPS-based stress control in parasites

Whereas mammals employ SIFI (UBR4/KCMF1) as a dedicated ISR-silencing module, parasites appear to rely on distributed ubiquitin-proteasome system (UPS) strategies (34). Apicomplexans, including *P. falciparum*, show functional Cullin-RING E3 ligases (PfSCF, PfCRL4) required for cell division, membrane integrity, and stress control (35). Parasite ERAD components such as HRD1 are essential for managing misfolded proteins (36). These proteostasis systems contribute to drug resistance, as artemisinin-resistant parasites display enhanced proteasomal degradation and improved stress tolerance compared with sensitive strains (20).

Trypanosomatids, such as *T. cruzi*, show diverse E3 ligase families with stage-specific expression. Comparative genomics indicates parasite-specific UPS adaptations, including E3 ligases, without clear eukaryotic homologs (37). Apicomplexans lack canonical HSF1 and instead use non-canonical AP2 factors for stress regulation (38). Despite sophisticated proteostasis, a single parasite equivalent of SIFI that couples stress detection to ISR termination has not been identified (38). This may reflect true evolutionary divergence which favors modular stress handling, or simply an underexplored area of parasite biology.

## 7 How parasites hijack host ISR/UPR pathways

Intracellular parasites modulate host stress responses to support survival and replication. Many activate host UPR pathways that protect infected cells from apoptosis, extending the intracellular niche. Mitochondria are also remodeled during infection, engaging defense programs that can restrict pathogen growth (39). The full spectrum of these triggers and molecular pathways, operatin at host levels, is detailed in **Table 2**.

### 7.1 *Plasmodium berghei*: tissue-dependent UPR outcomes

*P. berghei* triggers ER stress and UPR activation with context-dependent outcomes. In cerebral malaria models, all three UPR branches (PERK–eIF2α–ATF4, IRE1–XBP1s, ATF6) are activated in the brain, correlating with neuronal apoptosis (CHOP/caspase induction) and downregulation of protective chaperones (BiP, calreticulin) (8, 39). In contrast, during liver-stage infection, hepatocyte UPR activation via XBP1 and CREBH supports parasite development but loss of XBP1 splicing impairs parasite growth, consistent with a requirement for XBP1s-driven lipid synthesis during intrahepatic development (3).

### 7.2 *Trypanosoma cruzi* and *T. brucei*: ER stress, ISR, and parasite-specific death programs

*T. cruzi* infection could induce cardiac ER stress, following increase in BiP, PERK, eIF2α, ATF4, and CHOP (5). Inhibition of ER stress with 2-aminopurine reduces cardiomyopathy and decreases eIF2α phosphorylation (7). TcK1 has been linked to autophagy-dependent clearance of stress granules, though substrates beyond eIF2α remain unclear (30). In *T. brucei*, TcK3 has been reported to translocate from ER to nucleus under persistent stress, suppress transcription, and promote stress-induced parasite death via TATA-binding protein phosphorylation (30).

### 7.3 *Leishmania*: hormesis-like UPR tuning, antioxidant programs, and host survival

*L. infantum* induces mild UPR that confers a hormesis-like adaptive state, increasing resistance to subsequent ER stress and promoting survival through PI3K/Akt activation and caspase-3 inhibition (40). *L. amazonensis* activates IRE1-XBP1 and PERK/eIF2α/ATF4 via TLR2. XBP1s promotes IFN-β, induces HO-1, suppresses NO production, and promotes NF-κB p50/p50 dimerization to reduce iNOS transcription.Comparable XBP1s exploitation occurs in *P. berghei*–infected hepatocytes to support phospholipid synthesis for parasitophorous vacuole expansion (41, 42). LdeK1 can phosphorylate human eIF2α *in vitro*, raising the possibility of direct parasite kinase action on host translation. LiPERK (a K2 orthologue) is ER-localized and required for promastigote-to-amastigote differentiation and intracellular growth (43).

In *L. donovani* infection, host UPR activation delays apoptosis via PERK-dependent survival signaling and increased cIAP transcription, and inhibition of PERK phosphorylation under oxidative stress increases NO, decreases cIAP, and shifts infected cells toward apoptosis, reducing infection (44). ATF4 contributes to NRF2 and HO-1 induction, and ATF4 knockdown increases NO and reduces parasite burden, which can be rescued by antioxidants such as NAC (41, 42).

Protozoan parasites often display simplified UPR networks compared with metazoans, lacking canonical IRE1, XBP1/HAC1, ATF6, and ATF4 transcriptional machineries while retaining PERK/eIF2α translational control. Consistent with this, *L. donovani* is more sensitive than host macrophages to ER stressors such as DTT, suggesting that translational UPR alone offers less buffering than the full metazoan UPR (44). This vulnerability may represent a therapeutic opportunity via parasite-selective ER stress induction (3).

### 7.4 Autophagy as a parasite fitness module under stress

The ISR and autophagy are interconnected adaptive pathways that coordinate cellular responses to stress. Both systems share common upstream sensors and converge on overlapping transcriptional and post-translational programs to maintain proteostasis and organelle quality control (45). The ISR-autophagy crosstalk operates through multiple nodes. ATF4, the key transcriptional effector of the ISR, directly upregulates essential autophagy genes, including *LC3B* and *ATG5*, thereby coupling translational attenuation to enhanced autophagosome formation (46). Additionally, PERK-mediated eIF2α phosphorylation promotes selective autophagy of damaged organelles, particularly mitochondria (mitophagy), by modulating the expression of autophagy receptors and mitochondrial quality control proteins (47). Therefore, autophagy can modulate ISR signaling intensity by clearing stress-damaged organelles and protein aggregates, potentially reducing the initial trigger for ISR activation. Though this bidirectional interplay allows cells to fine-tune stress response, an acute ISR activation enhances autophagy to facilitate adaptation and survival. A chronic or dysregulated ISR can lead therefore to excessive autophagy and cell death (46, 48).

In parasites, this ISR-autophagy axis appears particularly critical for navigating developmental transitions across digenetic life cycles and hostile environments (49).

In *Leishmania spp*, autophagy increases during differentiation and under nutrient deprivation, oxidative stress, and drug exposure; while pharmacological inhibition markedly increases parasite mortality (48, 49).

ATG8 family proteins are essential autophagy markers that are critical for viability and virulence in Trypanosomatidae and Apicomplexa. In *Trypanosoma brucei*, disrupting TbATG7 or TbATG5 does not affect mammalian stages but impairs proliferation of the insect stage under starvation conditions. *Trypanosoma cruzi* requires starvation-induced autophagy involving TcATG8.1 for metacyclogenesis (50). In *Leishmania major*, autophagy markers increase during metacyclogenesis and in amastigotes, with autophagy-deficient mutants (LmATG5 or LmATG4.2 knockouts) showing impaired differentiation. Furthermore, ATG8 overexpression enhances stress resistance and macrophage infection capacity, while ATG8 deletion compromises parasite viability and completely blocks promastigote-to-amastigote differentiation and infectivity, both *in vitro* and *in vivo*. Additionally, ATG8 accumulates at damaged mitochondria in a ROS-dependent manner, indicating its involvement in mitochondrial quality control during stress conditions (51). Notably, disrupting LmATG4.1 does not affect autophagy, suggesting functional diversification among ATG proteins (51). In Apicomplexa, ATG8 proteins are more divergent and structurally simplified compared to those of Trypanosomatidae, yet they retain E3 ligase activity through non-covalent interactions rather than classical energy-intensive ubiquitin-like conjugation, representing a unique evolutionary adaptation (49).

These findings demonstrate that autophagy functions as a conserved adaptive mechanism in Trypanosomatidae and Apicomplexa, enabling parasites to successfully navigate developmental transitions between insect vectors and mammalian hosts. Importantly autophagy pathways intersect with ISR signaling to coordinate stress responses.

### 7.5 *Toxoplasma gondii*: ER stress-driven apoptosis and mitochondrial defenses

*T. gondii* can induce neural stem cell apoptosis via ER stress-dependent activation of CHOP, caspase-12, and JNK signaling, and ER stress inhibitors such as TUDCA block this phenotype (52). The virulence factor ROP18 contributes by phosphorylating ER-resident reticulon 1-C, associated with GRP78 acetylation. Despite lacking canonical ATF4/GCN4-like bZIP factors, Apicomplexa conserve stress-induced translational control via eIF2α, suggesting the existence of functionally analogous transcriptional regulators (6, 53).

During mitochondrial stress, mitochondria can shed their outer membrane via SPOT-like structures (Structures Positive for Outer Mitochondrial Membrane). Originally identified as a host safeguard remodeling response, some parasites like *T. gondii* may exploit this defense mechanism to facilitate the degradation of host factors that restrict parasite growth. During *T. gondii* infection, TgMAF1 binds the host import receptor TOM70, which in turn mediates a direct interaction between TgMAF1 and the OMM translocase SAM50. This TgMAF1/TOM70/SAM50 tripartite interaction induces disassembly of the mitochondrial intermembrane space bridging (MIB) complex, triggering SPOT formation and the consequent degradation of OMM proteins including mitofusin 1/2 (54). Host ATF4 can also mediate defense against *T. gondii* by rewiring mitochondrial one-carbon/folate metabolism, increasing mtDNA levels and restricting parasite access to folates required for dTMP synthesis (19).

## 8 Conclusion and outlook

The ISR is a key molecular interface in host-parasite interactions, but its net impact on disease outcomes remains highly context-dependent. Evidence supports parasite-driven exploitation of ISR/UPR signaling to promote survival and replication (43), yet robust ISR activation can also function as a host defense mechanism restricting parasite growth (5). These apparently conflicting findings likely reflect differences in parasite species, life-cycle stage, host-cell type, and the particular stress-response branch engaged.

From a translational perspective, the divergence in ISR architecture between parasites and hosts (including parasite-specific eIF2α kinases with unique regulation, and the apparent absence of SIFI-like silencing modules) offers opportunities for selective therapeutic intervention. Progress toward ISR-targeted strategies will require systematic dissection of (i) parasite ISR effectors across life-cycle stages, (ii) mechanisms of host–parasite ISR/UPR cross-talk, and (iii) therapeutic windows in which stress pathway modulation compromises parasite fitness while preserving host-cell function.
